# Axial torsion as a rare and unusual complication of a Meckel's diverticulum: a case report and review of the literature

**DOI:** 10.1186/1752-1947-5-118

**Published:** 2011-03-28

**Authors:** Ajai Seth, Jai Seth

**Affiliations:** 1 Brighton and Sussex Medical School, University of Sussex, Falmer, Brighton BN1 9PX, UK; 2 Department of Anatomy, School of Biomedical and Health Sciences, Guys Campus, King's College London, London, UK

## Abstract

**Introduction:**

In 1809, Johann Friedrich Meckel described the embryology of a small bowel diverticulum, which now bears his name. Meckel's diverticulum is the most common congenital abnormality of the gastrointestinal tract, with a prevalence ranging from 1% to 4% of the population. The majority are clinically silent and are incidentally identified at surgery or at autopsy. The lifetime risk of complications is estimated at 4%, with most of these complications occurring in adults. It is these cases that can cause problems for the clinician, as the diagnosis can be elusive and the consequences extremely serious.

**Case presentation:**

We present the case of a 68-year-old Caucasian man with axial torsion of a Meckel's diverticulum around its base, a rare complication. He presented with acute, severe abdominal pain, and a clinical diagnosis of perforated acute appendicitis was made. Laparotomy revealed a torted Meckel's diverticulum with distal necrosis and perforation, which was resected. His recovery was uncomplicated, and he was discharged to home six days post-operatively.

**Conclusion:**

Torsion is an extremely rare complication of Meckel's diverticulum. Its presentation can be elusive, and it can mimic a number of different, more common intra-abdominal pathologies. Imaging appears to be an unreliable diagnostic tool, and the diagnosis is usually made intra-operatively. Factors pre-disposing these patients to axial torsion of Meckel's diverticulum include the presence of mesodiverticular bands, a narrow base, excessive length, and associated neoplastic growth or inflammation of the diverticulum. The importance of searching for a diseased Meckel's diverticulum at laparotomy in appropriate circumstances is highlighted. Once identified, prompt surgical excision generally leads to an uncomplicated recovery.

## Introduction

Johann Friedrich Meckel first described the embryological origin of congenital diverticulum of the mid-gut in 1809 [1]. Meckel's diverticulum (MD) results from incomplete obliteration of the most proximal portion of the vitelline or omphalo-mesenteric duct occurring during weeks five to seven of fetal development [2]. It is thought that the terminal band represents an aberration in the developmental vitelline arteries, which in turn arise from the superior mesenteric or the ileocolic artery [3]. This fibrous band connects the diverticulum to the umbilicus [4]. Total failure of closure can result in an umbilical fecal fistula. Proximal ductal closure can lead to an umbilical sinus, whereas distal closure leads to MD [5]. Seventy-four percent of MD cases terminate with a blind distal end [5]. Histologically, all four intestinal layers are present within MD, and the mucosa may contain ectopic gastric, pancreatic, jejuna, or duodenal epithelium in up to 50% of specimens [5,6].

MD is invariably found on the anti-mesenteric border of the ileum, with 90% located within 90 cm of the ileocecal valve [2]. Its size is also variable, with the majority being short and wide-mouthed, with a mean length of 2.9 cm and a mean width of 1.9 cm, which is why it is sometimes called an ileal appendix [7]. Giant MD are defined as those larger than 5 cm, with one recorded specimen measuring 16 cm × 4 cm [2].

MD is more often diagnosed in men, as they are more prone to complications [1]. The most common childhood complication is rectal bleeding due to ileal peptic ulceration secondary to ectopic gastric mucosa [7,8]. Intestinal obstruction is the more common presentation in adults, caused by either intussusception or small bowel volvulus around a diverticular band anchored to the anterior abdominal wall. Other common complications include acute inflammation leading to perforation and hemorrhage [1]. Rarer complications include MD perforation with foreign bodies, strangulation in Littré's hernia, primary neoplasms, or vesicodiverticular fistulae [7,9]. Axial torsion of MD is an extremely rare complication [1,10]. Torsion of MD is the result of axial twisting around its base. This can occur around a persistent mesodiverticular band or with an absent band and a free-ended diverticulum. The exact mechanism for this is unclear. The degree of torsion varies and can compromise diverticular circulation, leading to necrosis and perforation [2].

## Case presentation

A 68-year-old Caucasian man presented to our hospital with acute, severe abdominal pain. An examination of the patient revealed that he was septic and had a distended abdomen with rebound tenderness in the hypogastrium and the right iliac fossa. His rectal examination was unremarkable. His blood test revealed a raised white cell count, 15.4 × 10^3^/μl, and a high C-reactive protein level at 208 mg/L. The patient had normal renal function and a normal hemoglobin level. An abdominal radiograph revealed dilated small bowel loops, and a clinical diagnosis of perforated acute appendicitis was made. No other pre-operative investigations were carried out, and following fluid resuscitation, a laparoscopy was performed.

**Figure 1:**
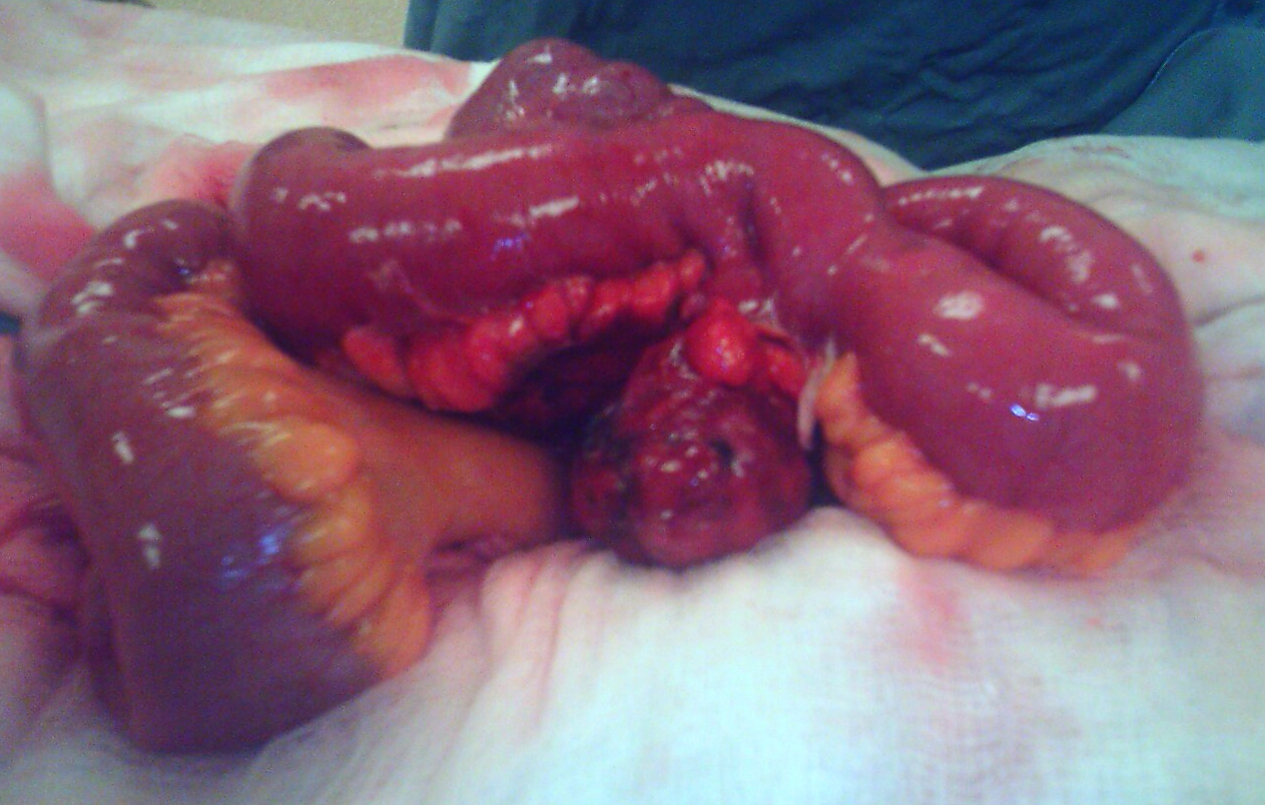
**The intra-operative finding of a torted Meckel's diverticulum with distal necrosis and perforation**. A torted Meckel's diverticulum with distal necrosis and perforation was found during surgery. The necrosed tip of the diverticulum was adherent to the adjacent mesentery with a normal appearance of the rest of the bowel and viscera. The twisted Meckel's diverticulum was resected along with an 8 cm flange of ileum.

Laparoscopy revealed purulent fluid within the pelvis. The appendix could not be visualized, but the peri-appendicular region appeared normal. The laparoscopy was converted to a laparotomy. Surgical exploration revealed a torted MD with distal necrosis and perforation. The necrosed tip of the diverticulum was adherent to the adjacent mesentery (Figure 1). The appendix, the rest of the bowel, and the viscera appeared normal. The twisted MD was resected along with an 8 cm flange of ileum that was encompassed within the vascular territory of the inflamed, unhealthy, and friable mesentery. An end-to-end seromuscular, single-layered anastomosis using a 4-0 synthetic absorbable suture, was performed to restore the continuity of the small bowel. Thorough washout of the peritoneal cavity was performed, and a pelvic drain was inserted. The patient's recovery was uncomplicated, and he was discharged to home six days post-operatively with routine follow-up.

## Discussion

This case report presents the unusual case of torsion of MD. By reviewing the previous literature, we aim to identify the possible etiology, main clinical features, appropriate investigations, and operative management associated with this variant.

The etiology of axial torsion of MD remains unclear. On the basis of the available literature, we have identified several risk factors. Although primary neoplasms arising within MD is rare, representing less than 1% of cases [11], they may be a potential risk factor. A large review of 1605 cases of complications of MD identified only 24 cases [9]. A variety of benign and malignant histological types have been reported, including leiomyoma, fibroma, hemangioma, neurofibroma, carcinoid tumor, adenocarcinoma, fibrosarcoma, and leiomyosarcoma [11]. Benign lesions within MD, such as lipomas, have also been recognized as a potential cause of torsion [12]. Complications associated with this presentation include intussusception, with the tumor as the lead point, mechanical intestinal obstruction, volvulus, inflammation, and axial torsion [13]. Fibrous vitelline bands may exist and connect the MD to the abdominal wall, increasing the chance of its torting [5]. An increase in diverticular length and the size of the base is an important predisposition for all types of complications [14]. The larger and longer the MD, the greater the risk of torsion [2]. This risk is increased further if the MD has a narrow neck and is less likely to tort around a wider neck [14,15].

Pain is always a presenting feature of a torted MD but is more frequently localized to the right lower quadrant [16]. Pain duration may range from 24 hours of colicky episodic pain to three years of intermittent pain. The patient described by Tan and Zheng [14] was discovered to have a giant MD, which was thought to be causing repeated episodes of torsion and ischemia during this time. The pre-operative diagnosis of MD is rarely considered [4]. Common incorrect diagnoses have included appendicitis [17], small bowel obstruction, cholecystitis, or an amoebic liver abscess. The latter case, reported by Webster [18], represents a case of an MD that was fixed within a sub-phrenic location. The mobility of MD can therefore determine its clinical features, which vary with its position within the abdomen. Therefore, it can also make radiological investigation confusing. When clinically suspected appendicitis is insufficiently inflamed, further abdominal exploration is important [16].

Because of its various forms of presentation and unreliable imaging, torsion of MD is frequently misdiagnosed. Special investigations appear to have little value in the diagnosis of acute MD complications. Abdominal radiographs are usually normal but may reveal an ileus or perforation [4]. Less common radiographic appearances have included gas-filled diverticula being mistaken for emphysematous cholecystitis, intussusception in infants, and even a report of MD containing calculi simulating gallstones [8]. Ultrasound may exclude intussusception, which can avoid unnecessary interventions such as attempts at reduction by the use of enemas. The MD appears similar to the bowel, with a layered wall; however, when torted, it mimics a cystic, tube-like, non-peristaltic structure [8]. The major difference is acute appendicitis. A larger size and a location far from the ileocecal region would favor the diagnosis of axial MD torsion [8]. Computed tomographic scans may also be misleading, as described in case reports of a torted MD's being mistaken for a loculated cystic pelvic mass [3,19].

Appendicitis is the main pre-operative diagnosis, while other diagnoses include small bowel obstruction, acute cholecystitis, and liver abscess [2,18,20]. Macroscopic intra-operative observations have been reported as torsion, ischemic appearance, hemorrhagic, gangrenous, and perforated with purulent peritonitis [10]. A further observation from the previous literature is that the degree of torsion is inversely proportional to the viability of the MD. In cases where there is a greater degree of torsion, there is also a greater vascular compromise to the MD [2]. This risks infarction and perforation, which are associated with greater morbidity. The post-operative period may be complicated by intra-abdominal abscess or either clinical or microscopic evidence of lower gastrointestinal bleeding [10,20].

The management of symptomatic MD is surgical resection. A wedge resection of the MD is generally carried out, and occasionally some ileum is resected by end-to-end anastomosis [7]. Diverticulectomy for MD found incidentally has been criticized, as a potential 800 asymptomatic resections are required to prevent a single patient from complications [5]. However, if the MD is left intact, any fibrous bands attached to it must be excised to prevent any future torsion or obstruction [5].

## Conclusion

In summary, this case report describes a patient with torsion of MD. Imaging appears to be unreliable in the detection of torted MD, and the diagnosis is usually made intra-operatively. Major risk factors for torsion appear to include an increased size of the MD with a narrow base, potentially compromising blood supply and leading to gangrene, the presence of a fibrous mesodiverticular band, and the rare presence of neoplasm. The importance of suspecting MD pathology in the differential diagnosis and its confirmation at laparotomy has been highlighted. Once identified, prompt surgical excision generally leads to an uncomplicated recovery.

## Consent

Written informed consent was obtained from the patient for publication of this case report and any accompanying images. A copy of the written consent is available for review by the Editor-in-Chief of this journal.

## Competing interests

The authors declare that they have no competing interests.

## Authors' contributions

JS was the surgical senior house officer who diagnosed the case. AS performed the literature search. Both authors were involved in the writing of the report.
